# Hydroisomerization of *n*-Butane over Platinum-Promoted Cesium Hydrogen Salt of 12-Tungstophosphoric Acid

**DOI:** 10.3390/ma2042319

**Published:** 2009-12-14

**Authors:** Yanyong Liu, Makoto Misono

**Affiliations:** 1 Biomass Technology Research Center, National Institute of Advanced Industrial Science and Technology, AIST Tsukuba Central 5, 1-1-1 Higashi, Tsukuba, Ibaraki 305-8565, Japan; 2 Professor Emeritus, University of Tokyo, Bunkyu-ku, Tokyo 113-8656, Japan

**Keywords:** hydroisomerization, *n*-butane, platinum, heteropolyacid, bifunctional catalyst

## Abstract

The hydroisomerization of *n*-butane was carried out in a fixed-bed gas-flow reactor over Pt-promoted Cs_2.5_H_0.5_PW_12_O_40_ (denoted as Cs2.5). Two kinds of catalysts, a direct impregnation of Pt on Cs2.5 (denoted as Pt/Cs2.5), as well as a mechanical mixture of Pt/Al_2_O_3_ and Cs2.5 (denoted as Pt/Al_2_O_3_+Cs2.5), were used for the hydroisomerization. Pt/Al_2_O_3_+Cs2.5 showed a higher stationary activity than Pt/Cs2.5 because the Pt particles supported on Al_2_O_3_ were much smaller than those supported on Cs2.5. The initial activity decreased with increasing H_2_ pressure over Pt/Al_2_O_3_+Cs2.5. This indicates that the hydroisomerization of *n*-butane over Pt/Al_2_O_3_+Cs2.5 proceeded through a bifunctional mechanism, in which *n*-butane was hydrogenated/dehydrogenated on Pt sites and was isomerized on acid sites of Cs2.5. For the hydroisomerization of *n*-butane over Pt/Al_2_O_3_+Cs2.5 the hydrogenation/dehydrogenation on Pt sites is a limiting step at a low Pt loading and the isomerization on solid acid sites is a limiting step at a high Pt loading. During the reaction, hydrogen molecules were dissociated to active hydrogen atoms on Pt sites, and then the formed active hydrogen atoms moved to the solid acid sites of Cs2.5 (spillover effect) to eliminate the carbonaceous deposits and suppress the catalyst deactivation. Because Cs2.5 has suitably strong and uniformly-distributed solid acid sites, Pt/Al_2_O_3_+Cs2.5 showed a higher stationary activity than Pt/Al_2_O_3_+H-ZSM-5 and Pt/Al_2_O_3_+SO_4_/ZrO_2_ for the hydroisomerization of *n*-butane at a low H_2_ pressure.

## 1. Introduction

Polyoxometalates, including heteropolyoxometalates and isopolyoxometalates, can be used as excellent catalysts for both acid-catalyzed reactions and oxidation reactions [1,2,3,4,5,6,7,8,9,10,11,12,13,14,15]. Among them, 12-tungstophosphoric acid (H_3_PW_12_O_40_), a heteropolyacid with Keggin structure, has strong acidity and is widely used for many important catalytic reactions [1,2,3]. However, H_3_PW_12_O_40_ is limited by its low surface area (<10 m^2^ g^–1^), which hinders its application in gas-flow fixed-bed reactions. Introduction of large counter cations (such as K, NH_4_, Cs, and so on) into H_3_PW_12_O_40_ greatly increases the surface area. In particular, a partial substitution of Cs^+^ for H^+^ of H_3_PW_12_O_40_ substantially enhances its activity for many acid-catalyzed reactions [1,2,3]. Cs_2.5_H_0.5_PW_12_O_40_ (abbreviated as Cs2.5) is usually the most active solid acid for many reactions among the various Cs_x_H_3–x_PW_12_O_40_ catalysts due to its strong acidity (similar to H_3_PW_12_O_40_) and large surface area (>200 m^2^ g^–1^) [1,2,3]. 

*n*-Butane is an important industrial chemical which can be obtained from the petroleum industry and Fischer–Tropsch synthesis process. The skeletal hydroisomerization of *n*-butane to isobutane is a large-scale industrial process. Isobutane is utilized in the butene alkylation to isooctane as well as in the produce of MTBE (methyl *tert*-butyl ether) or ETBE (ethyl *tert*-butyl ether). All of these products are well known as non-leaded high octane gasoline additives. The hydroisomerization of *n*-butane occurs through carbenium cation intermediates, which requires the presence of strong acid in the system. Bifunctional catalysts containing metals and solid acids are promising for the hydroisomerization of *n*-butane. The metal sites provide the hydrogenation-dehydrogenation function and the acid sites provide the isomerization function. In generally, Pt is the most effective metal catalyst, and thus the development of highly active solid acid catalysts is an important task for designing bifunctional catalysts in the hydroisomerization of *n*-butane.

The commercial process for the hydroisomerization of *n*-butane is performed using the Pt/Cl–Al_2_O_3_ catalyst [16,17]. This process requires a continuous addition of toxic and corrosive chloride additives to restore the chloride species that leached slowly during the reaction. Therefore, it is urgent to develop a solid acid catalyst without chloride for the industrial process of *n*-butane hydro-isomerization. Heteropolyacids [18,19,20,21,22,23], solid superacids (SO_4_/ZrO_2_, WO_x_/TiO_2_, *etc.*) [24,25,26,27,28,29,30,31,32], and acidic zeolites (H-ZSM-5, H-Beta, *etc.*) [33,34,35,36,37,38,39] have been investigated for the hydroisomerization of *n*-butane.

How to combine the Pt catalyst with the solid acid catalyst is an interesting subject in catalyst design. For preparing bifunctional catalysts, although the direct support of the Pt on the solid acid by impregnation is a universal method, the mechanical mixing Pt/Al_2_O_3_ or Pt/SiO_2_ with the solid acid is a unique method. As early as fifty years ago, a mechanical mixture of Pt/SiO_2_ and aluminum silicates had been used for investigating the reaction mechanism of saturated hydrocarbons isomerization over bifunctional catalysts [40]. In the recent years, mechanical mixed catalysts have been of interest for the hydroisomerization of *n*-butane because they have some advantages compared with the directly impregnated catalysts. These advantages include the strong mechanical strength, the high molding ability, and so on [27,28,35]. We have found that the mechanically mixed catalyst Pt/Al_2_O_3_+Cs2.5 showed a higher activity and a higher stability than those of the directly impregnated catalyst Pt/Cs2.5 for the hydroisomerization of *n*-pentane and *n*-hexane [5,6,7]. In the present study, we investigated the catalytic performance of Pt-promoted Cs2.5 catalysts for the hydroisomerization of *n*-butane and also compared Pt-promoted Cs2.5 catalysts with Pt-promoted SO_4_/ZrO_2_ and Pt-promoted H–ZSM-5 for the hydroisomerization of *n*-butane.

## 2. Experimental Section

### 2.1. Catalyst Syntheses

H_3_PW_12_O_40_ was purchased from Wako Pure Chemical Company. Cs_2.5_H_0.5_PW_12_O_40_ (abbreviated as Cs2.5) was prepared by titrating an aqueous solution of H_3_PW_12_O_40_ (0.08 mol L^–1^) with an aqueous solution of Cs_2_CO_3_ (0.12 mol L^–1^), as described in detail in the literature [41,42]. 

Pt/Cs2.5 was prepared from aqueous solutions H_2_PtCl_6_, H_3_PW_12_O_40_ and Cs_2_CO_3_ as reported in the literature [18]. To an aqueous solution of H_3_PW_12_O_40_ (0.08 mol L^–1^, 44 mL), an aqueous solution of H_2_PtCl_6_ (0.04 mol L^–1^, 16 mL) was added dropwise at 323 K to obtain a yellow solution. Then the aqueous solution of Cs_2_CO_3_ (0.12 mol L^–1^, 36 mL) was added dropwise to the solution at 323 K at a rate of about 0.6 mL min^–1^. The resulting colloidal solution was evaporated to dryness at 323 K. The molar ratio of Pt:Cs^+^:PW_12_O_40_ was 0.18:2.5:1.0, where the amount of Pt in Pt/Cs2.5 corresponds to 1.0 wt%.

Pt/Al_2_O_3_ was prepared by the impregnation of Al_2_O_3_ (JRC-ALO-4, 167 m^2^ g^–1^) in an aqueous solution of H_2_PtCl_6_. After drying at 373 K for 24 h, the sample was calcined at 773 K for 3 h in air. The loading of Pt for Pt/Al_2_O_3_ was 2.0 wt%.

The mechanically mixed catalyst of Pt/Al_2_O_3_ with Cs2.5 (abbreviated as Pt/Al_2_O_3_+Cs2.5) was prepared as follows: after grinding the mixture of 2.0 wt% Pt/Al_2_O_3_ with Cs2.5 (mass ratio = 1:1) in a mortar for 30 min, the powder was pressed into a disk at 40 kg cm^–2^ and then sieved to 24–60 mesh. Thus the Pt loading in Pt/Al_2_O_3_+Cs2.5 was 1.0 wt%.

SO_4_/ZrO_2_ (abbreviated as SZ) was prepared using a method reported in the literature [43]. Zr(OH)_4_, which was obtained by the hydrolysis of ZrOCl_2_ with NH_4_OH, was treated with an aqueous solution of H_2_SO_4_ (1 N). After filtering out the liquid (H_2_SO_4_ solution), the resulting solid (SO_4_^2–^-Zr(OH)_4_) was calcined at 773 K for 3 h in air to form SO_4_/ZrO_2_. 

H-ZSM-5 (abbreviated as HZ) was obtained from Na-ZSM-5 (Tosoh Corporation, HSZ-820 NAA, SiO_2_/Al_2_O_3_ = 23.2, surface area: 322 m^2^ g^–1^) by the ion-exchange method. Na–ZSM-5 was treated with an aqueous solution of NH_4_NO_3_ (1 N) to form NH_4_-ZSM-5, followed by drying at 373 K for 24 h. NH_4_-ZSM-5 was calcined at 773 K for 3 h to form H–ZSM-5.

The mechanically mixed catalysts of Pt/Al_2_O_3_ with SZ (abbreviated as Pt/Al_2_O_3_+SZ) and Pt/Al_2_O_3_ with HZ (abbreviated as Pt/Al_2_O_3_+HZ) were prepared using a method similar to that of Pt+Cs2.5. After grinding the mixture of 2.0 wt% Pt/Al_2_O_3_ with SZ or HZ (mass ratio = 1:1) in a mortar for 30 min, the powder was pressed into a disk at 40 kg cm^–2^ and then sieved to 24–60 mesh. Thus the Pt loadings in either Pt/Al_2_O_3_+SZ or Pt/Al_2_O_3_+HZ were 1.0 wt%.

### 2.2. Characterization

Scanning electron microscope (SEM) observations were carried out using a Hitachi S-3400N instrument with an EDX. Pt metal surface and Pt particle size were measured by a H_2_ adsorption method. The H_2_ uptake was estimated by the extrapolation to zero pressure of the linear part of the isotherms. The difference between the total amount of adsorbed hydrogen (H_tot_) and the reversible part of adsorbed hydrogen (H_rev_) gave the irreversible part of adsorbed hydrogen (H_irr_), which was used for calculating the Pt metal surface and Pt particle size. Temperature-programmed desorption of ammonia (NH_3_-TPD) was observed using a BELCAT-B automatic monitor equipped with a TCD and a mass spectrometer for ammonia species detection. A part of a 0.05 g aliquot of the sample was pretreated at 673 K for 1 h under He flow (50 mL min^–1^). After the temperature was decreased to 373 K, ammonia was adsorbed onto the surface, followed by evacuation for 1 h at 373 K to eliminate the weakly adsorbed ammonia. Then, NH_3_-TPD was carried out from 373 K to 973 K (8 K min^–1^).

### 2.3. Catalytic Reaction

The hydroisomerizations of *n*-butane was performed in a fixed-bed quartz tubular reactor (φ: 8 mm) at 573 K under atmospheric pressure. In a typical reaction conditions, the total flow rate was20 mL min^–1^, the catalyst amount was 1 g, and the feed gas contained 10% *n*-C_4_H_10_, 10% H_2_, and80% N_2_. The catalysts were pretreated in a flow of H_2_ (60 mL min^–1^) at 573 K for 1 h before reaction. During reaction, the products were analyzed with an on-line FID GC (Hitachi GC-163) equipped with an Al_2_O_3_/KCl fused silica capillary column.

## 3. Results and Discussion 

### 3.1. Hydroisomerization of n-Butane over Various Catalysts

**Figure 1:**
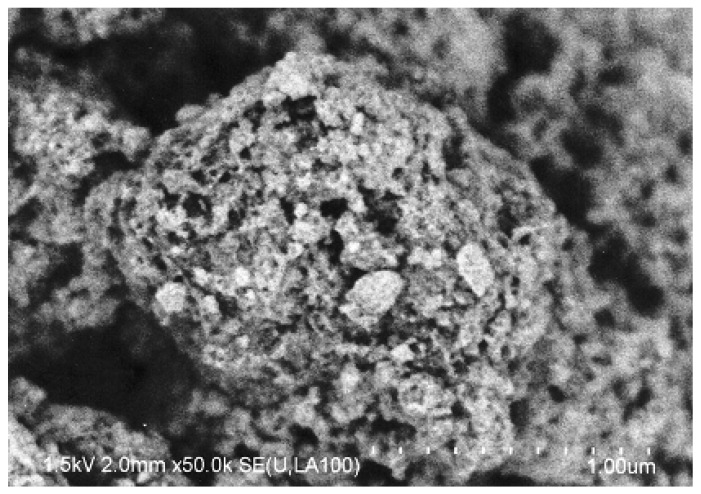
SEM picture of Pt/Al_2_O_3_+Cs2.5 (mass ratio = 1:1) after grinding for 30 min.

Figure 1 shows the SEM picture of the mixture of Pt/Al_2_O_3_ with Cs2.5 (mass ratio = 1:1) after grinding in a mortar for 30 min. 

The size of Pt/Al_2_O_3_ particles was about 0.5 μm. The Cs2.5 became small powder particles adhered to the Pt/Al_2_O_3_ particles due to the low mechanical strength of Cs2.5. The Pt/Al_2_O_3_ particles and the Cs2.5 particles contacted closely with each other. Moreover, the mechanical strength was greatly strengthened after pressing the mixture into a disk at 40 kg cm^–2^ for use as a catalyst.

Table 1 lists conversion and selectivity for the hydroisomerization of *n*-butane at 573 K over various catalysts. The data at 5 min could be regarded as the initial catalytic performance and the data at 5 h could be regarded as the stable catalytic performance over each catalyst. 

**Table 1:** Catalytic hydroisomerization of *n*-butane over various catalysts at 573 K.

Catalyst	Time on stream	Conv. (%)	Selectivity (%)					
			C_1_	C_2_	C_3_	*i*-C_4_	C_4_^=^	C_5+_
Pt/Al_2_O_3_	5 min	4.2	9.7	12.6	9.3	66.2	0	2.2
	5 h	2.8	8.6	9.2	8.1	71.3	0	2.8
Cs2.5	5 min	23.5	2.6	6.8	9.6	76.5	0.8	3.7
	5 h	9.2	1.8	5.1	7.3	82.3	0.7	2.8
Pt/Cs2.5	5 min	66.1	1.9	3.2	4.7	88.4	0.7	1.0
	5 h	42.2	1.6	2.5	4.1	90.3	0.6	0.9
Pt/Al_2_O_3_+Cs2.5	5 min	70.3	1.2	2.4	3.9	91.2	0.6	0.6
	5 h	64.8	0.8	2.0	3.3	92.5	0.5	0.8

*n*-Butane: 0.1 atm; H_2_: 0.1 atm; N_2_: 0.8 atm; total flow rate: 20 mL min^–1^.

Pt/Al_2_O_3_ showed a low conversion (2.8%) after 5 h on stream, which indicates that catalyst acidity is indispensable for the hydroisomerization of *n*-butane. Cs2.5 showed an initial conversion of 23.5% and an initial selectivity to isobutane of 76.5%. The solid acid Cs2.5 could catalyze the hydroisomerization of *n*-butane even without Pt [44]. However, the conversion after 5 h on stream over Cs2.5 was low (9.2%), due to the severe deactivation. The initially white Cs2.5 catalyst became black after reaction at 573 K for 5 h, indicating that carbonaceous deposits were formed on the catalyst surface, covering the acid sites of Cs2.5. This is the reason for the deactivation of the Cs2.5 catalyst. On the other hand, Pt/Cs2.5 showed an initial conversion of 66.1% and a stable conversion of 42.2%. The synergy between Pt and Cs2.5 was great, because both the initial conversion and the stable conversion were remarkably improved by introducing Pt in the solid catalyst Cs2.5. Moreover, Pt/Al_2_O_3_+Cs2.5 exhibited a higher stable conversion (64.8%) and a higher stable selectivity to isobutane (92.5%) than those over Pt/Cs2.5 for the hydroisomerization of *n*-butane after 5 h on stream. On the other hand, the mixture of Pt/Cs2.5 and Al_2_O_3_ just showed a similar performance (not shown in Table 1) to that of Pt/Cs2.5 for the hydroisomerization of *n*-butane. Thus the method for combining Pt with Cs2.5 is important for increasing the catalytic activity of bifunctional catalysts in the hydroisomerization of *n*-butane.

**Scheme 1:**
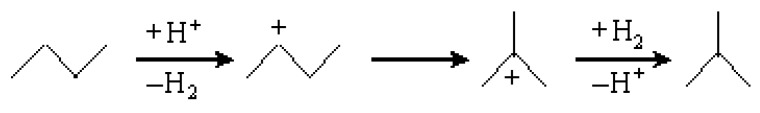
Mechanism of *n*-butane hydroisomerization over a Brønsted acid catalyst.

Scheme 1 shows the mechanism of *n*-butane hydroisomerization over a Brönsted acid catalyst. Since a heteropolyacid is a kind of typical Brönsted acid [1], it is very probable that the hydroisomerization of *n*-butane over Cs2.5 also takes place *via* this mechanism [44]. At first, a *sec*-butyl carbenium cation was formed by a step of proton addition, followed by a step of H_2_ elimination. Then, the *sec*-butyl carbenium cation was transformed to a *tert*-carbenium cation by a shift of the methyl group. Finally, the *tert*-carbenium cation captured a H_2_ molecule and eliminated a proton to form an isobutane molecule. All of these steps were carried out on the Brønsted acid sites.

**Scheme 2:**
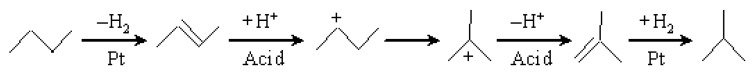
Mechanism of *n*-butane hydroisomerization over a bifunctional catalyst.

Scheme 2 shows the mechanism of *n*-butane hydroisomerization over a bifunctional catalyst containing metal and heteropolyacid [18,19]. At first, the *n*-butane molecule eliminated a H_2_ molecule to form a *n*-butene molecule on the Pt sites. Then, the formed *n*-butene molecule moved to the solid acid sites to form a *sec*-butyl carbenium cation by obtaining a proton. Then, the *sec*-butyl carbenium cation was transformed to a *tert*-carbenium cation by a shift of the methyl group on the acid sites. Then, the *tert*-carbenium cation eliminated a proton to form an isobutene molecule. Finally, the isobutene molecule moved to the Pt sites to form an isobutane molecule by a process of H_2_ addition.

**Figure 2:**
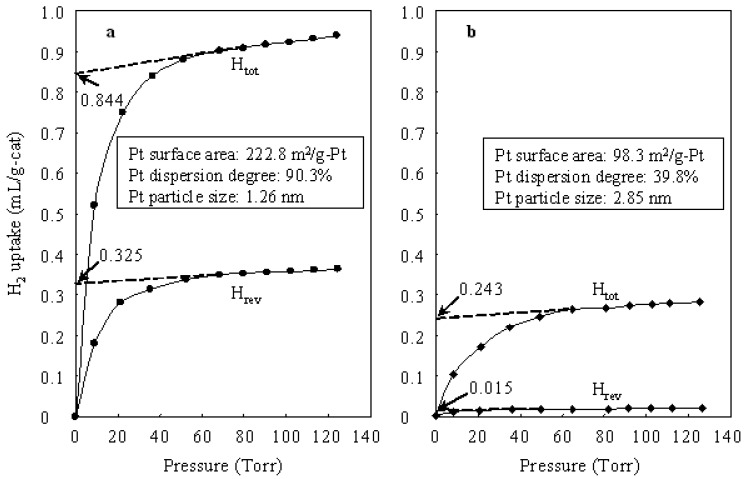
(a) H_2_ uptake by Pt/Al_2_O_3_+Cs2.5 at 298 K. (b) H_2_ uptake by Pt/Cs2.5 at 298 K.

As a result, although the carbenium cation is a key intermediate in the hydroisomerization of *n*-butane over either a heteropolyacid catalyst or a bifunctional catalyst containing Pt and heteropolyacid, the path for forming the carbenium intermediate over a bifunctional catalyst is different from that over a heteropolyacid catalyst. Because the rate of formation of a carbenium species by adding proton to *n*-butene is much faster than that by adding proton to *n*-butane, in the hydroisomerization of *n*-butane the bifunctional catalysts (Pt/Cs2.5 and Pt/Al_2_O_3_+Cs2.5) showed much higher conversions than those observed over the monofunctional heteropolyacid catalyst Cs2.5 (Table 1). Figure 2 shows H_2_ uptake by Pt/Al_2_O_3_+Cs2.5 and Pt/Cs2.5 at 298 K. The H_2_ uptake was used as for calculating the Pt surface area, Pt dispersion degree, and Pt particle size for the samples. The total H_2_ uptake contains the reversible H_2_ uptake (physical absorption) and the irreversible H_2_ uptake (chemical absorption). The irreversible H_2_ uptake at 0 torr could be obtained from the total H_2_ uptake at 0 torr and the reversible H_2_ uptake at 0 torr. The obtained irreversible H_2_ uptake at 0 torr was used for calculating Pt surface area and Pt dispersion degree of each sample. As shown in Figure 2, the Pt surface area and Pt dispersion degree over Pt/Al_2_O_3_+Cs2.5 were much larger than those over Pt/Cs2.5. Thus Al_2_O_3_ is a good support for Pt as comparison with Cs2.5. The high Pt dispersion degree gave Pt/Al_2_O_3_+Cs2.5 a higher selectivity for isobutane than that over Pt/Cs2.5 in the hydroisomerization of *n*-butane (Table 1).

Although the catalysts prepared by directly supporting Pt on solid acids (using the impregnation method) are usually used for the hydroisomerization of *n*-alkanes, the mechanical mixtures of solid acids with Pt/SiO_2_ or Pt/Al_2_O_3_ have also been applied for the hydroisomerization of *n*-alkanes for several purposes. Firstly, a mechanical mixture of Pt/SiO_2_ with aluminum silicates had been used for investigating the mechanism of *n*-alkanes hydroisomerization over Pt-promoted acid catalysts [40]. Because Pt sites and solid acid sites achieved their functions independently, the mechanical mixed catalyst showed a catalytic activity similar to that seen over the directly impregnated catalysts [40]. Secondly, because the Pt-supported zeolites have low mechanical strength and poor molding properties, the mechanical mixtures of acidic zeolites with Pt/SiO_2_ or Pt/Al_2_O_3_ have been used to increase the mechanical strength and the molding ability of the bifunctional catalysts [33,35,45]. Thirdly, in the cause of solid superacid SO_4_^2-^-ZrO_2_, the directly supported Pt/SO_4_^2-^-ZrO_2_ catalyst is not a bifunctional catalyst due to the interaction of Pt with the sulfur on the SO_4_^2-^-ZrO_2_ surface, while the mechanical mixed catalyst Pt/Al_2_O_3_+SO_4_^2-^-ZrO_2_ is a bifunctional catalyst [27]. Therefore, the mechanical mixed catalysts Pt/SiO_2_+SO_4_^2-^-ZrO_2_ and Pt/Al_2_O_3_+SO_4_^2-^-ZrO_2_ showed high catalytic performances for the hydroisomerization of *n*-butane [27,28,46]. Fourthly, in the case of the heteropolyacid Cs_2.5_H_0.5_PW_12_O_40_, the mechanical mixed catalyst Pt/Al_2_O_3_+Cs2.5 showed a higher activity than that of the impregnated catalyst Pt/Cs2.5 for the hydroisomerization of *n*-pentane and *n*-hexane [5,6,7]. In the present study, we found that Pt/Al_2_O_3_+Cs2.5 showed a higher catalytic performance for the hydroisomerization of *n*-butane in comparison with Pt/Cs2.5. The directly supported catalyst Pt/Cs2.5 had a low Pt surface area and a low Pt dispersion degree, probably because Pt^2+^ interacted with PW_12_O_40_^3–^ during the impregnation process.

### 3.2. Deactivation of Various Catalysts in the Hydroisomerization of n-Butane 

**Figure 3:**
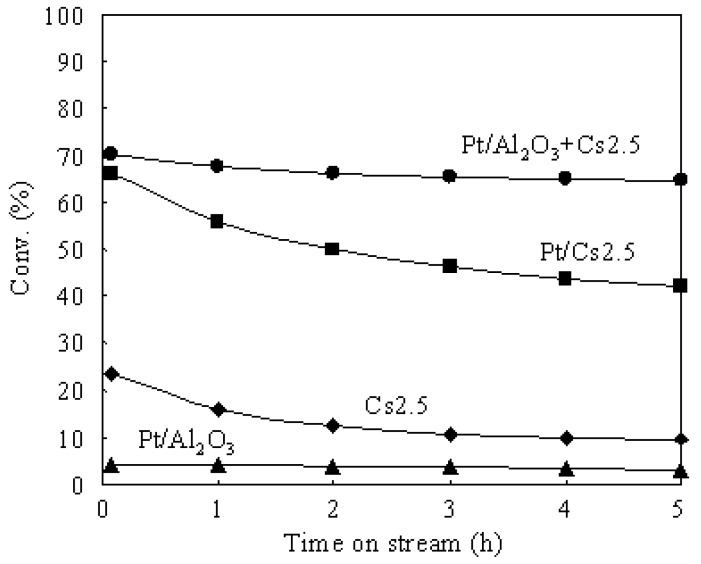
Time courses of the hydroisomerization of *n*-butane over various catalysts at 573 K.

Figure 3 shows the time courses of *n*-butane hydroisomerization over various catalysts at 573 K. Pt/Al_2_O_3_ showed a very low conversion due to the lack of strong acid sites. Thus Pt/Al_2_O_3_ can barely be used as an independent catalyst for the hydroisomerization of *n*-butane. The presence of strong acid sites in the catalyst is indispensable for the hydroisomerization of *n*-butane. Cs2.5 showed an initial conversion (after 5 min on stream) of 23.5%, but the conversion decreased to 9.2% after 5 h on stream. The carbonaceous deposits which formed by the polymerization of alkene intermediates and by-products (such as *n*-butene, *iso*-butene, and so on) covered the solid acid sites and caused a serious deactivation of the Cs2.5 catalyst. The carbonaceous deposits are hydrocarbons with large molecular weights and high ratios of C to H. When Pt was introduced into Cs2.5 (*i.e.*, Pt/Cs2.5 and Pt/Al_2_O_3_+Cs2.5), the deactivation was greatly repressed in the hydroisomerization of *n*-butane. Pt catalyzes the hydrogenation of the carbonaceous deposits (covered acid sites) by supplying hydrogen. In concrete, the remarkable effect of Pt in suppressing the catalyst deactivation was brought about by the activated hydrogen, which were formed on Pt, transferred to Cs2.5, and utilized to remove the carbonaceous deposits [5,6]. Pt/Al_2_O_3_+Cs2.5 showed a higher catalytic stability than that of Pt/Cs2.5 for the hydroisomerization of *n*-butane because the Pt dispersion degree in Pt/Al_2_O_3_+Cs2.5 was much higher than that in Pt/Cs2.5 (Figure 2).

The amount of the carbonaceous deposits on the used catalyst could be calculated by a temperature-programmed oxidation (TPO) method [47,48,49]. After the reaction was carried out over each catalyst for 5 h at 573 K, the reactor was cooled to room temperature in flowing N_2_ gas. The catalyst was then treated in air flow (1.5 L h^−1^) by increasing the temperature at 2.5 K min^−1^ to change the carbonaceous deposits to CO_2_. The formed CO_2_ could be detected by a TCD GC. 

**Figure 4:**
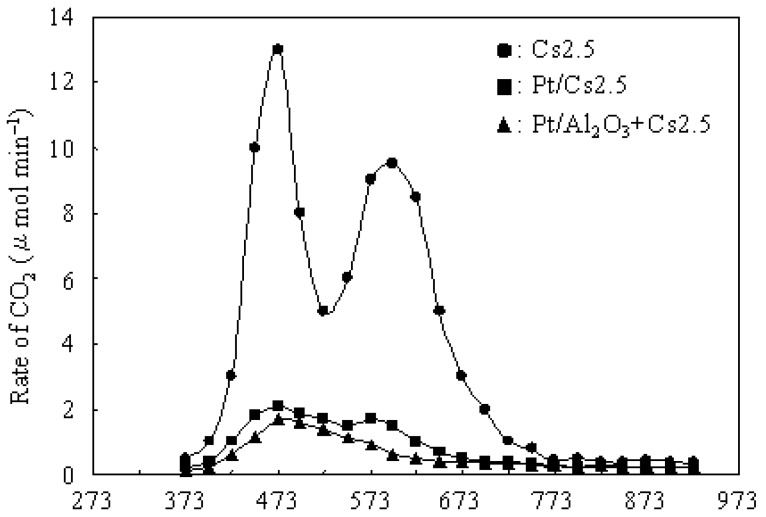
Dependence of the rate of CO_2_ (formed from carbonaceous deposits) on the calcination temperature over various catalysts after 5 h on stream at 573 K.

Figure 4 shows the dependence of the rate of CO_2_ (formed from carbonaceous deposits) on the calcination temperature over various catalysts after 5 h on stream at 573 K. 

An integration of the rate of CO_2_ formation gave the amount of total CO_2_, from which the amount of the carbonaceous deposits (after 5 h on stream at 573 K) were calculated as 4.5, 0.6, and 0.4 wt% on Cs 2.5, Pt/Cs2.5, and Pt/Al_2_O_3_+Cs2.5, respectively. Moreover, according to the amount of CO_2_ and H_2_O formed in the TPO measurement for various catalysts after 5 h on stream at 573 K, the H/C ratios were calculated as about 0.3, 0.6, and 0.7 for the carbonaceous deposits on Cs 2.5, Pt/Cs2.5, and Pt/Al_2_O_3_+Cs2.5, respectively. Two peaks with the highest rate at around 473 and 598 K were observed from the plot of Cs2.5. In general, the peak at lower temperature could be regarded as “soft coke” and the peak at the higher temperature could be regarded as “hard coke” [50,51]. Pt/Cs2.5 deposited both “soft coke” and “hard coke” on the surface, but the amount deposited on the Pt/Cs2.5 surface was much lower than that on the Cs2.5 surface. Thus Pt/Cs2.5 showed a much higher stability than that over Cs2.5 at 573 K (Figure 3). Further, the amount of carbonaceous deposit on Pt/Al_2_O_3_+Cs2.5 (0.4 wt.%) was lower than that on Pt/Cs2.5 (0.6 wt.%) after 5 h on stream. In the TPO profile of Pt/Al_2_O_3_+Cs2.5, the peak of “hard coke” at 573–598 K was very small and just became a shoulder peak for the peak of “soft coke” at low temperature. Therefore, Pt/Al_2_O_3_+Cs2.5 showed a very high stability for the hydroisomerization of *n*-butane (Figure 3).

### 3.3. Hydroisomerization of n-Butane over the Pt/Al_2_O_3_+Cs2.5 Catalyst

**Figure 5:**
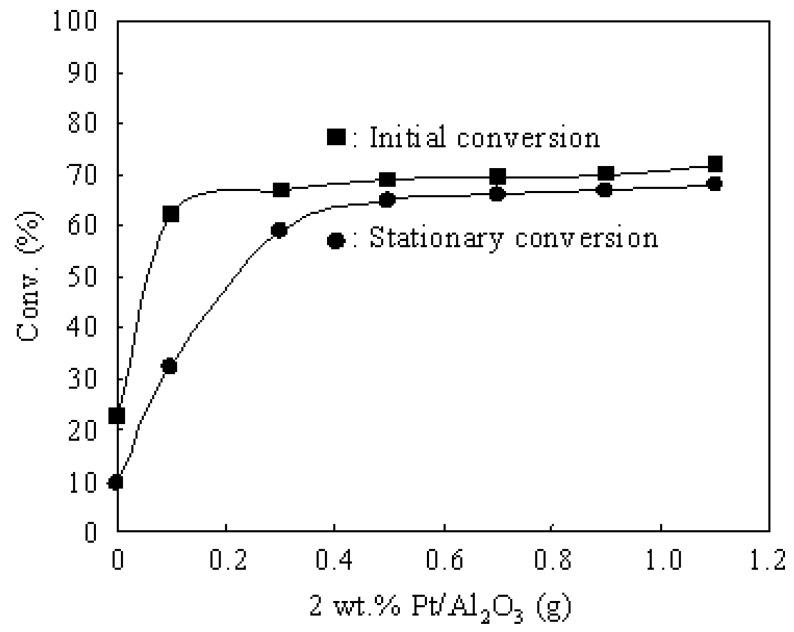
Effect of Pt amount in the Pt/Al_2_O_3_+Cs2.5 catalyst for the hydroisomerization of *n*-butane at 573 K. **(■)** Initial conversion. **(●)** Stationary conversion. Cs2.5: 0.5 g. Reaction conditions: *n*-butane: 0.1 atm; H_2_: 0.1 atm; N_2_: 0.8 atm; total flow rate: 20 mL min^–1^.

Figure 5 shows the effect of Pt amount in the Pt/Al_2_O_3_+Cs2.5 catalyst for the hydroisomerization of *n*-butane at 573 K. Either the pressure of *n*-butane or the pressure of H_2_ was 0.1 atm. The initial conversion greatly increased when a small amount of 2 wt.% Pt/Al_2_O_3_ was added to Cs2.5, but it almost remained at a constant value when the amount of 2 wt.% Pt/Al_2_O_3_ was more than 0.1 g. On the other hand, 0.5 g of 2 wt.% Pt/Al_2_O_3_ was necessary for suppressing the deactivation to make the stationary conversion close to the initial conversion. Thus Pt has two effects in the hydroisomerization of *n*-butane. Firstly, Pt achieves a hydrogenation-dehydrogenation function, which greatly improves the initial conversion over Pt/Al_2_O_3_+Cs2.5. It seems that 0.1 g of 2 wt.% Pt/Al_2_O_3_ is enough for increasing the initial conversion. Therefore, the hydrogenation-dehydrogenation on Pt sites is a limiting step when the amount of Pt/Al_2_O_3_ is less than 0.1 g in Pt/Al_2_O_3_+Cs2.5, and the isomerization on Cs2.5 sites is a limiting step when the amount of Pt/Al_2_O_3_ is more than 0.1 g in Pt/Al_2_O_3_+Cs2.5. Therefore, the speed of hydrogenation-dehydrogenation on Pt is very fast and a small amount of Pt can achieve the hydrogenation-dehydrogenation function. Secondly, Pt plays an important role for suppressing the catalyst deactivation and for maintaining the catalytic stability for the hydroisomerization of *n*-butane. The deactivation can not be completely suppressed with a small amount of Pt/Al_2_O_3_ (Figure 5). It needs a relatively large amount of 2 wt.% Pt/Al_2_O_3_ (about 0.5 g) to eliminate the catalyst deactivation over Pt/Al_2_O_3_+Cs2.5 for the hydroisomerization of *n*-butane.

**Figure 6:**
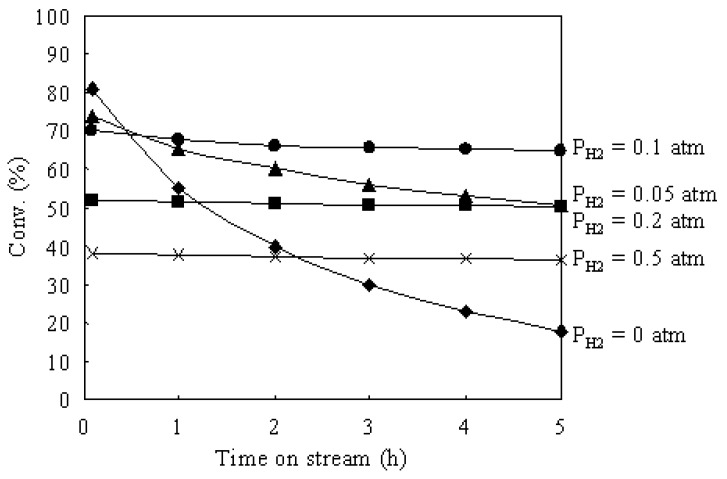
Time courses of the hydroisomerization of *n*-butane over Pt/Al_2_O_3_+Cs2.5 at 573 K under various H_2_ pressures. *N*-butane: 0.1 atm; N_2_: balance; total flow rate: 20 mL min^–1^.

Figure 6 shows the time courses of the hydroisomerization of *n*-butane over Pt/Al_2_O_3_+Cs2.5 at 573 K under various H_2_ pressures. The partial pressure of *n*-butane was 0.1 atm and the partial pressure of H_2_ was changed from 0 to 0.5 atm. Under a N_2_ atmosphere (P_H2_ = 0), the initial conversion was very high, but the deactivation was serious. With increasing H_2_ pressure, the deactivation was gradually suppressed, but the initial conversion decreased. Thus H_2_ has two effects in the hydroisomerization of *n*-butane: decreasing the initial conversion and suppressing the catalyst deactivation. Actually, H_2_ plays two roles in the hydroisomerization of *n*-butane over bufunctional catalysts. One role of H_2_ is the hydrogenation of isobutene to isobutane on Pt sites (the last step in Scheme 2). According to Scheme 2, the first step is the dehydrogenation of *n*-butane to *n*-butene for the hydroisomerization of *n*-butane over a bifunctional catalyst. Under a high H_2_ partial pressure, the equilibrium of the dehydrogenation step shifts to *n*-butane, which causes the decrease of the concentration of *n*-butene intermediates in the reaction system. As a result, because the presence of H_2_ is not favorable for the dehydrogenation step in the hydroisomerization of *n*-butane (the first step in Scheme 2), the initial conversion decreases with increasing H_2_ pressure over Pt/Al_2_O_3_+Cs2.5. Another role of H_2_ in the hydroisomerization of *n*-butane over bifunctional catalysts is the suppression of catalyst deactivation. As discussed above, the carbonaceous deposits that formed on the catalyst surface cause the catalyst deactivation. H_2_ molecules can form active H atoms on Pt sites during the reaction. The formed active H atoms shift (spillover) to the acid sites and hydrogenate the carbonaceous deposits on the acid sites of Cs2.5. Therefore, the catalyst deactivation can be suppressed by increasing H_2_ pressure in the hydroisomerization of *n*-butane over Pt/Al_2_O_3_+Cs2.5.

**Figure 7:**
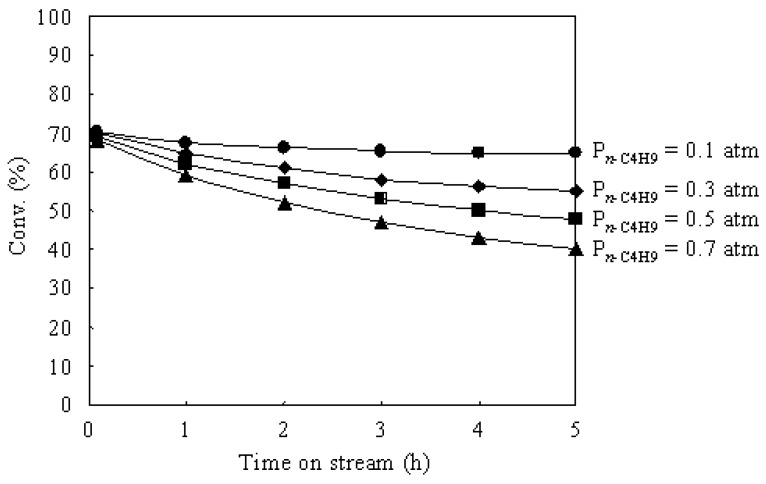
Time courses of the *n*-butane hydroisomerization over Pt/Al_2_O_3_+Cs2.5 at 573 K under various *n*-butane pressures. H_2_: 0.1 atm; N_2_ balance; total flow rate: 20 mL min^–1^.

Figure 7 shows the time courses of the *n*-butane hydroisomerization over Pt/Al_2_O_3_+Cs2.5 at 573 K under various *n*-butane pressures. The partial pressure of H_2_ was 0.1 atm and the partial pressure of *n*-butane was changed from 0.1 to 0.7 atm.

The initial conversions over Pt/Al_2_O_3_+Cs2.5 showed almost the same values under various *n*-butane pressures. This implies that the initial rate is proportional to the *n*-butane pressure (*i.e.*, first order in *n*-butane). Thus the “bimolecular mechanism” (by alkylation-cracking a C_8_ intermediate) is not important for the hydroisomerization of *n*-butane over Pt/Al_2_O_3_+Cs2.5 [6,43]. On the other hand, the conversion after 5 h on stream decreased with increasing the *n*-butane pressure. The increase of *n*-butane pressure means the decrease in the ratio of H_2_ to *n*-butane in the feed gas, which caused the catalyst deactivation during the reaction. Therefore, it needs a high H_2_ pressure to maintain the catalyst stability for the *n*-butane hydroisomerization over Pt/Al_2_O_3_+Cs2.5 under a high partial pressure of *n*-butane.

### 3.4. Comparison of Various Bifunctional Catalysts for the Hydroisomerization of n-Butane

Table 2 lists the results obtained in the hydroisomerization of *n*-butane with various bifunctional catalysts. We compared the Pt/Al_2_O_3_+Cs2.5 catalyst with the catalysts of Pt/Al_2_O_3_+SZ and Pt/Al_2_O_3_+HZ in this study because both Pt-promoted solid super acid SZ (SO_4_^2-^-ZrO_2_) and Pt-promoted acidic zeolite HZ (H-ZSM-5) have been reported as effective catalysts for the hydroisomerization of *n*-butane [24,25,26,27,28,29,30,31,32,33,34,35].

**Table 2:** Comparison of various bifunctional catalysts for the hydroisomerization of *n*-butane.

Catalyst	Time on stream	Conv. (%)	Selectivity (%)					
			C_1_	C_2_	C_3_	*i*-C_4_	C_4_^=^	C_5+_
Pt/Al_2_O_3_+Cs2.5	5 min	70.3	1.2	2.4	3.9	91.2	0.6	0.6
	5 h	64.8	0.8	2.0	3.3	92.5	0.5	0.8
Pt/Al_2_O_3_+SZ	5 min	77.6	7.7	10.6	14.3	61.2	1.3	4.8
	5 h	20.5	4.6	7.2	10.1	71.7	1.0	5.2
Pt/Al_2_O_3_+HZ	5 min	39.7	2.7	3.1	5.7	87.1	0.9	0.5
	5 h	32.2	1.8	3.3	4.6	88.7	1.1	0.4

Reaction temperature: 573 K; *n*-butane: 0.1 atm; H_2_: 0.1 atm; N_2_: 0.8 atm; catalyst: 1 g; total flow rate: 20 mL min^–1^.

**Figure 8:**
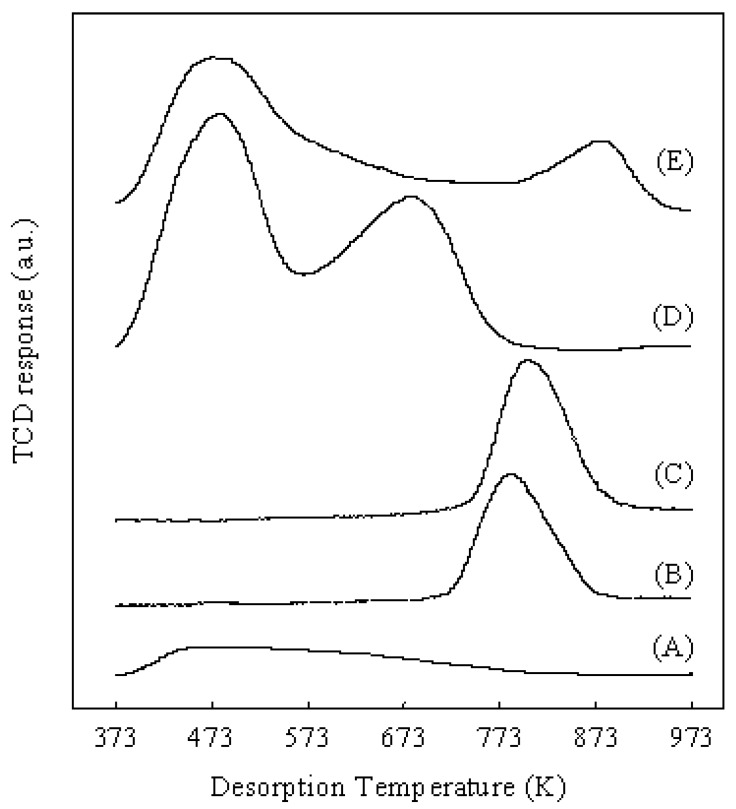
NH_3_-TPD profiles of various catalysts. (A): Pt/Al_2_O_3_; (B): Pt/Cs2.5; (C): Pt/Al_2_O_3_+Cs2.5; (D): Pt/Al_2_O_3_+HZ; (E): Pt/Al_2_O_3_+SZ.

Pt/Al_2_O_3_+Cs2.5, Pt/Al_2_O_3_+SZ and Pt/Al_2_O_3_+HZ had the same Pt dispersion because the Pt metal was supported on Al_2_O_3_ for all three catalysts. Therefore, the difference of solid acids (Cs2.5, SZ, and HZ) determined the difference of catalytic performances for the hydroisomerization of *n*-butane over various catalysts. As shown in Table 2, Pt/Al_2_O_3_+Cs2.5 showed the highest conversion (64.8%) and the highest selectivity for isobutane (92.5%) among the various catalysts after 5 h on stream. Pt/Al_2_O_3_+SZ showed the highest initial conversion (77.6%), but the conversion rapidly decreased to 20.5% after 5 h on stream. Moreover, the selectivity for isobutane over Pt/Al_2_O_3_+SZ was low. Pt/Al_2_O_3_+HZ showed lower conversion and selectivity than those over Pt/Al_2_O_3_+Cs2.5. As a result, Pt/Al_2_O_3_+Cs2.5 is the best catalyst for the hydroisomerization of *n*-butane at a low H_2_ pressure (0.1 atm). Brønsted acid sites in Cs2.5 contributed to the acid function of Pt/Al_2_O_3_+Cs2.5 for the hydroisomerization of *n*-butane since heteropolyacids are pure Brønsted acids without Lewis acid sites [1]. Figure 8 shows the NH_3_-TPD profiles of various catalysts. The NH_3_-TPD is a powerful tool for measuring the acidic strength of a solid acid. The NH_3_ molecules desorbed from the weak acid sites at low temperatures and desorbed from the strong acid sites at high temperatures.

**Figure 9:**
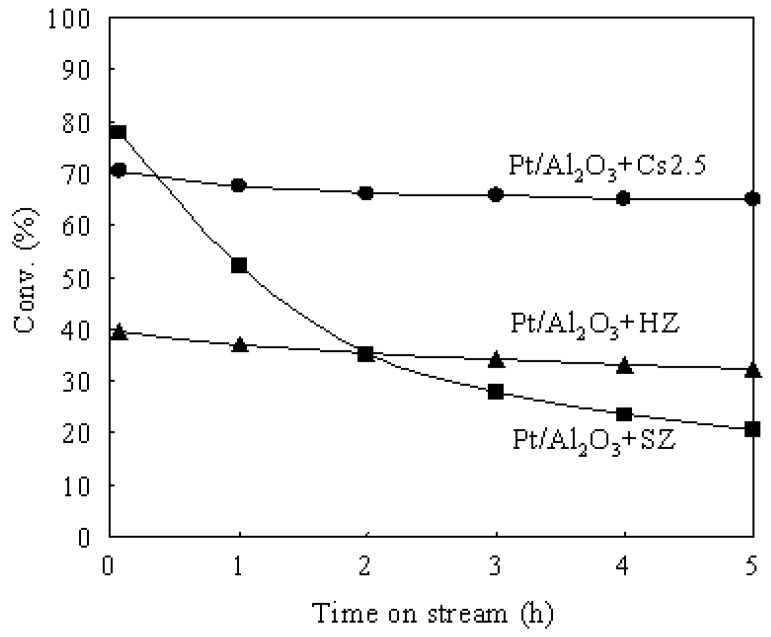
Time courses various bifunctional catalysts for the hydroisomerization of *n*-butane at 573 K. **(●)** Pt/Al_2_O_3_+Cs2.5, **(■)** Pt/Al_2_O_3_+SZ, **(▲)** Pt/Al_2_O_3_+HZ.

Pt/Al_2_O_3_ showed a weak and broad peak at about 473 K in the NH_3_-TPD profile, indicating that the acid sites in Pt/Al_2_O_3_ are very weak. Although Pt/Cs2.5 and Pt/Al_2_O_3_+Cs2.5 showed the peaks with similar shape in the NH_3_-TPD profiles, the maximum temperature of NH_3_ desorption from Pt/Cs2.5 was slightly lower than that from Pt/Al_2_O_3_+Cs2.5. Thus the acidic strength of Pt/Cs2.5 was slightly weaker than that of Pt/Al_2_O_3_+Cs2.5. This is the reason that Pt/Al_2_O_3_+Cs2.5 showed a higher initial conversion than that over Pt/Cs2.5 (Figure 3). The decrease of the acid strength of Pt/Cs2.5 probably because the ion exchange of Pt^2+^ with H^+^ in Cs_2.5_H_0.5_PW_12_O_40_ occurred in the impregnation stage of the synthesis of Pt/Cs2.5. For a solid acid, the strongest acid sites provide its main character for the acid-catalyzed reactions. The peak at the maximum temperature in the NH_3_-TPD profile corresponds to the strongest acid sites and thus it determines the acidic strength of a solid acid. According to the peak position at the maximum temperature in the NH_3_-TPD profile of various samples (Figure 8), the acidic strength of various catalysts was in an order of Pt/Al_2_O_3_+SZ > Pt/Al_2_O_3_+Cs2.5 > Pt/Cs2.5 > Pt/Al_2_O_3_+HZ > Pt/Al_2_O_3_. Pt/Al_2_O_3_+HZ showed two peaks at around 473 K and 673 K in the NH_3_-TPD profile, while Pt/Al_2_O_3_+SZ showed two peaks at around 473 K and 873 K in the NH_3_-TPD profile. These results indicate that both Pt/Al_2_O_3_+HZ and Pt/Al_2_O_3_+SZ have two types of acid sites: weak acid sites and strong acid sites. The hydroisomerization of *n*-butane occurs through carbenium cation intermediates which are easy to be formed on the strong acid sites. Thus the strong acid sites provide the main effect for the catalytic performances over Pt/Al_2_O_3_+HZ and Pt/Al_2_O_3_+SZ. However, the weak acid sites also catalyze the reaction although the reaction rate is low. Because the products formed from the strong acid sites and the weak acid sites are different, the broadly distributed acid sites caused the decrease of the selectivity for isobutane over Pt/Al_2_O_3_+HZ and Pt/Al_2_O_3_+SZ (Table 2). On the other hand, either Pt/Cs2.5 or Pt/Al_2_O_3_+Cs2.5 showed only one peak in the NH_3_-TPD profile, implying that the strength of the acid sites were distributed uniformly on the surfaces of Pt/Cs2.5 and Pt/Al_2_O_3_+Cs2.5. The uniform acid strength gave Pt/Cs2.5 and Pt/Al_2_O_3_+Cs2.5 high selectivity for isobutane in the hydroisomerization of *n*-butane (Table 1). Figure 9 shows the time courses of various bifunctional catalysts for the hydroisomerization of *n*-butane at 573 K. Either the pressure of *n*-butane or the pressure of H_2_ was 0.1 atm.

For a bifunctional catalyst in the hydroisomerization of *n*-alkanes, the balance between acid and metal is very important for obtaining the optimum performance [52,53]. The acid strength is an important factor for controlling the activity and the selectivity in the hydroisomerization of *n*-alkanes. Strong acids usually exhibit high activity but also usually show low selectivity and serious deactivation. 

As shown in Figure 9, the initial conversion was in the order Pt/Al_2_O_3_+SZ > Pt/Al_2_O_3_+Cs2.5 > Pt/Al_2_O_3_+HZ. This order coincided with the order of the acid strength of the various catalysts. Moreover, Pt/Al_2_O_3_+SZ showed a serious deactivation under a low H_2_ pressure (0.1 atm) and the activity after 5 h on stream was low. A high H_2_ pressure is need for the hydroisomerization of *n*-butane over the Pt-promoted SZ (SO_4_^2-^-ZrO_2_) catalysts [25,26,27,28,29,30,31]. Actually, the deactivation of Pt/Al_2_O_3_+SZ could be suppressed under a high H_2_ pressure of 0.7 atm. Moreover, because the conversion over Pt/Al_2_O_3_+Cs2.5 decreased with increasing H_2_ pressure (Figure 6), Pt/Al_2_O_3_+SZ showed a higher stationary conversion than that over Pt/Al_2_O_3_+Cs2.5 under a high H_2_ pressure of 0.7 atm. On the contrary, Pt/Al_2_O_3_+Cs2.5 showed a higher stationary conversion than that over Pt/Al_2_O_3_+SZ under a low H_2_ pressure of 0.1 atm (Figure 9). Thus Pt/Al_2_O_3_+Cs2.5 is a good catalyst under low H_2_ pressures and Pt/Al_2_O_3_+SZ is a good catalyst under high H_2_ pressures for the hydroisomerization of *n*-butane. In comparison with Pt/Al_2_O_3_+SZ, using Pt/Al_2_O_3_+Cs2.5 as a catalyst for the hydroisomerization of *n*-butane decreases the cost of the process (because H_2_ is much more expensive than N_2_) and improves the safety of the operation (because a gas stream containing a large amount of H_2_ is very dangerous).

## 4. Conclusions

By introducing Pt in Cs_2.5_H_0.5_PW_12_O_40_, the activity and the selectivity for isobutane in the hydroisomerization of *n*-butane were greatly increased. Because the Pt surface area and Pt dispersion degree of Pt/Al_2_O_3_ are much larger than those of Pt/Cs2.5, the mechanical mixed catalyst Pt/Al_2_O_3_+Cs2.5 showed a higher stationary conversion than that over the directly supported catalyst Pt/Cs2.5. Moreover, Pt/Al_2_O_3_+Cs2.5 showed a higher initial conversion than that over Pt/Cs2.5 because the acid strength of Pt/Cs2.5 was lower than that of Pt/Al_2_O_3_+Cs2.5 (due to the ion exchange of Pt^2+^ with H^+^ in the stage of impregnation). Comparing with Pt/Al_2_O_3_+HZ, Pt/Al_2_O_3_+Cs2.5 showed a higher activity (due to the stronger acid strength of Cs2.5) and a higher selectivity for isobutane (due to the uniformly-distributed acid sites on Cs2.5). Pt/Al_2_O_3_+SZ showed the highest initial conversion among various catalysts, but the activity decreased rapidly under a low H_2_ pressure (due to the excessively strong acidity of SO_4_^2-^-ZrO_2_). As a result, Pt/Al_2_O_3_+Cs2.5 is an excellent catalyst for the hydroisomerization of *n*-butane under a low H_2_ pressure because Pt/Al_2_O_3_ has highly dispersed Pt particles and Cs_2.5_H_0.5_PW_12_O_40_ has properly strong and uniformed distributed solid acid sites.
